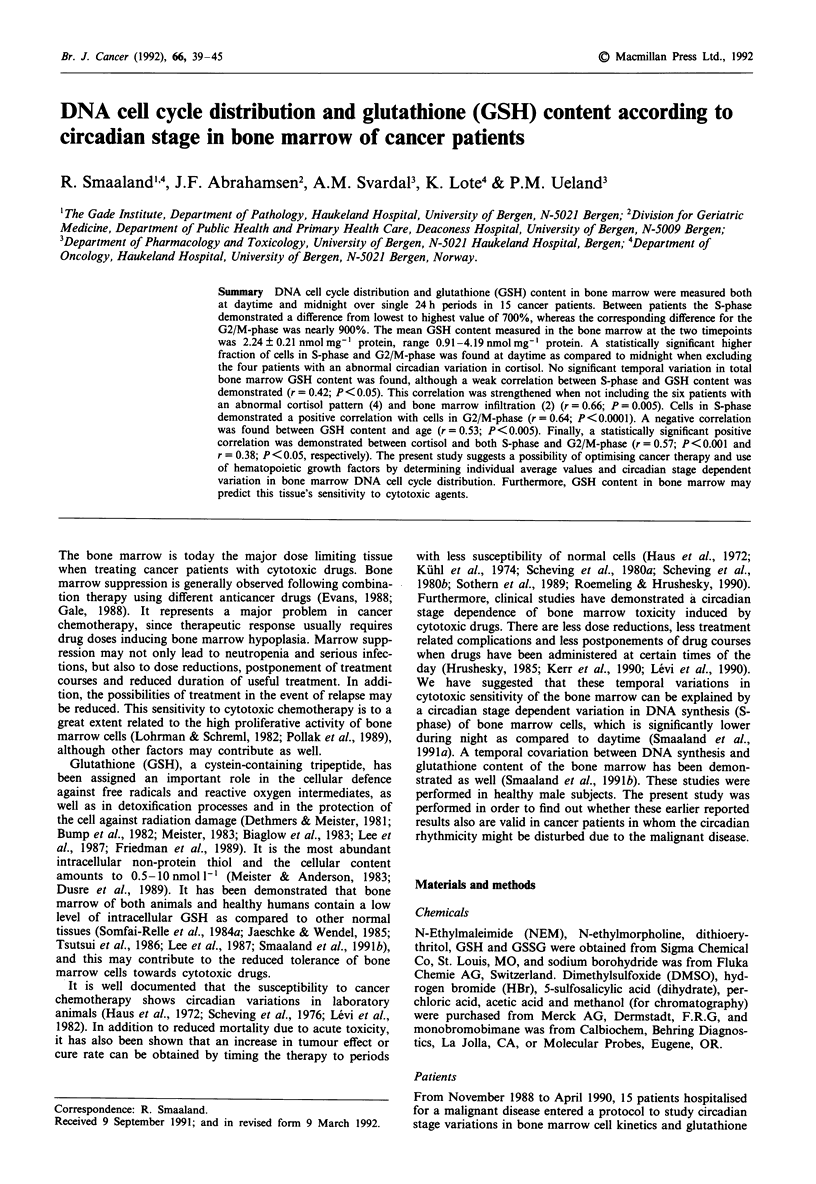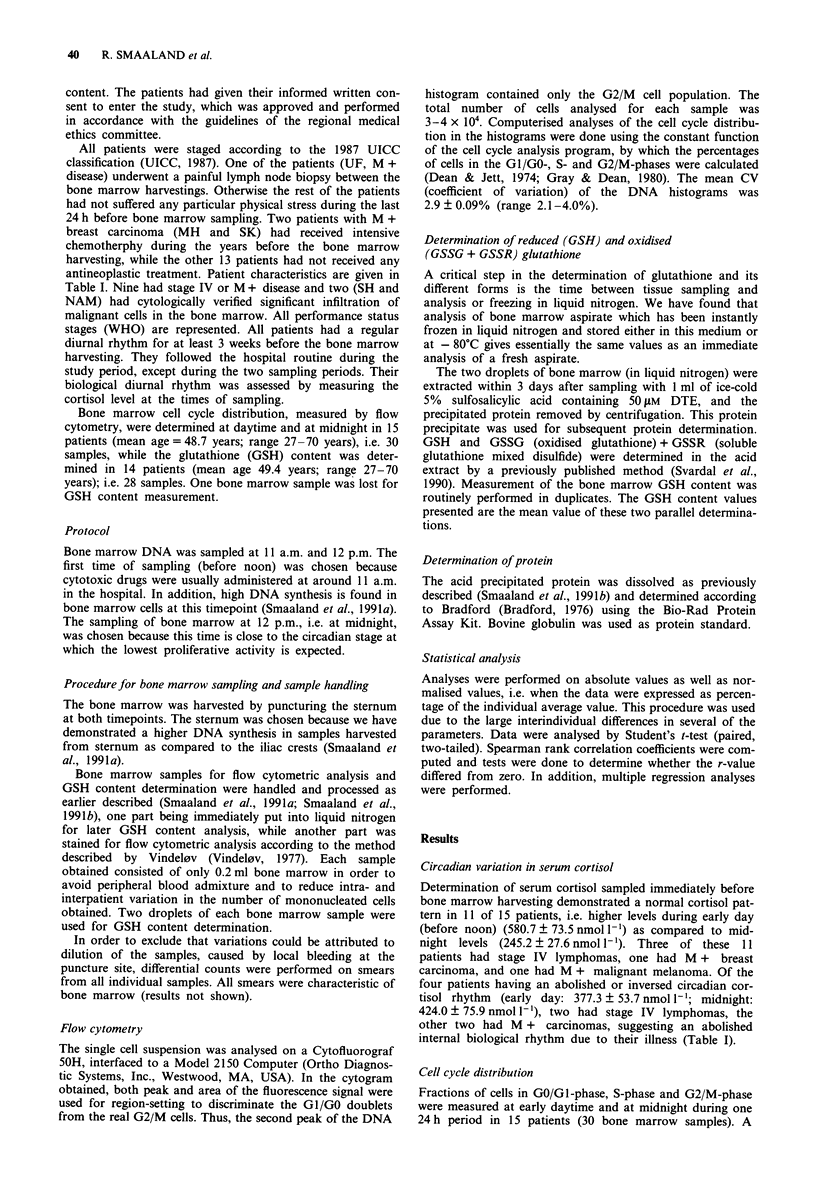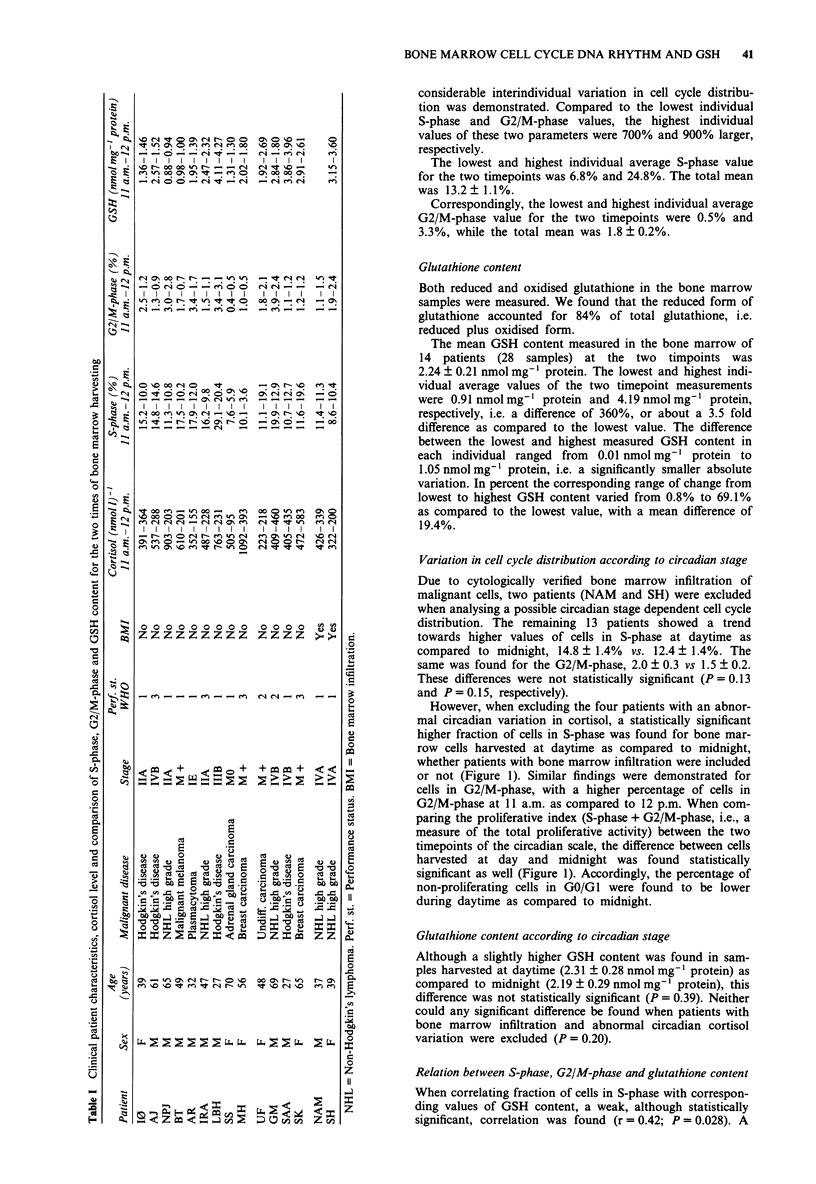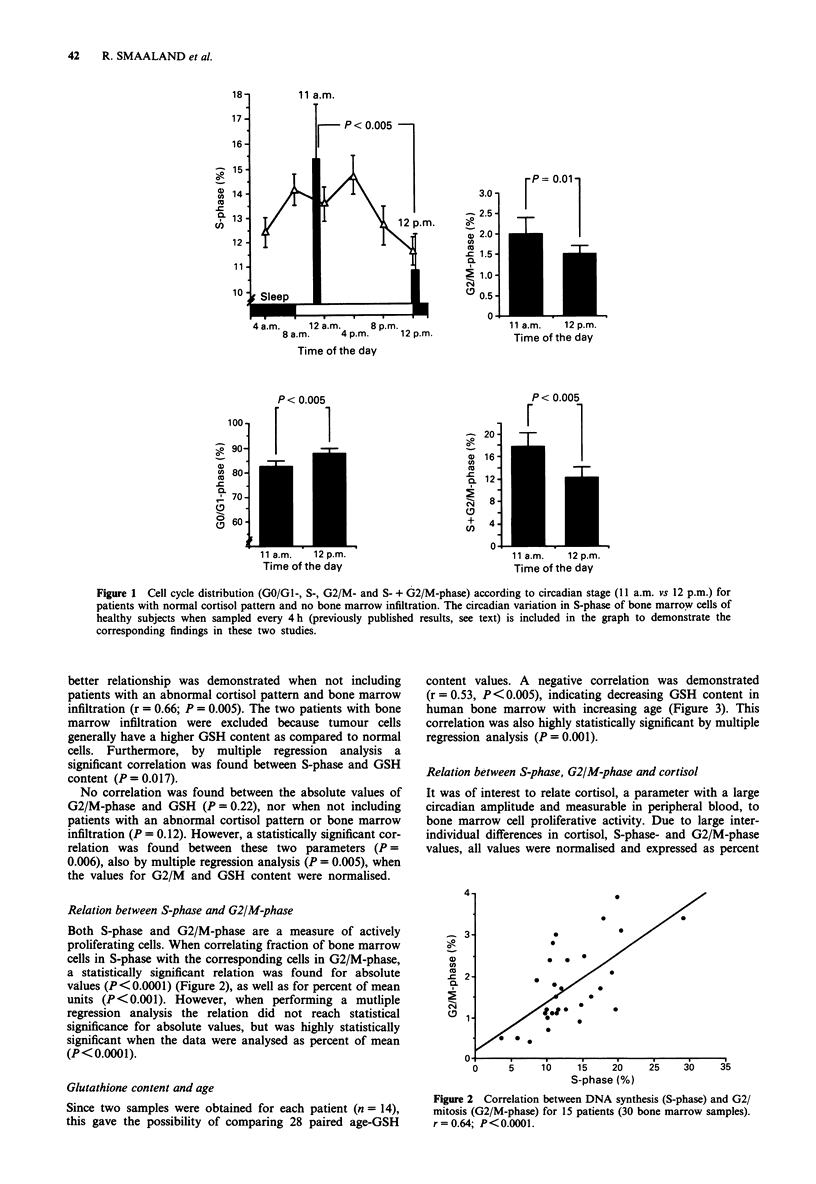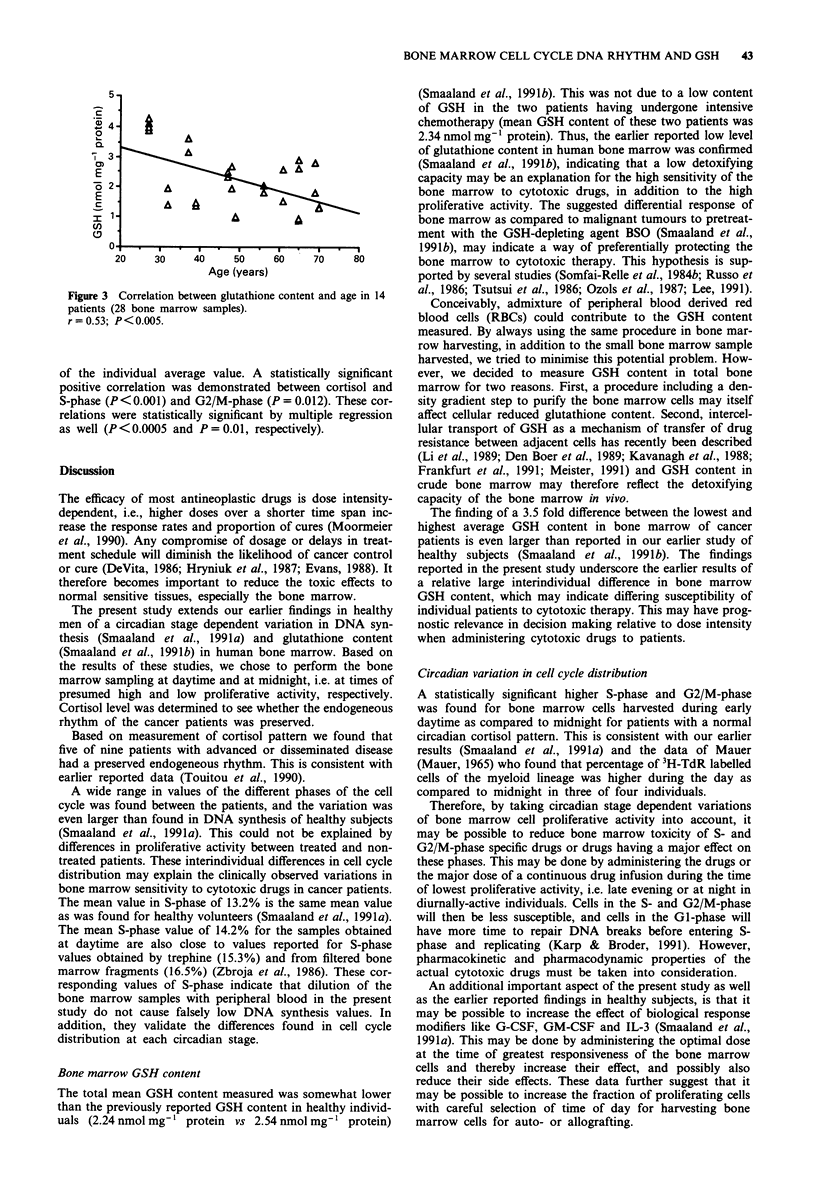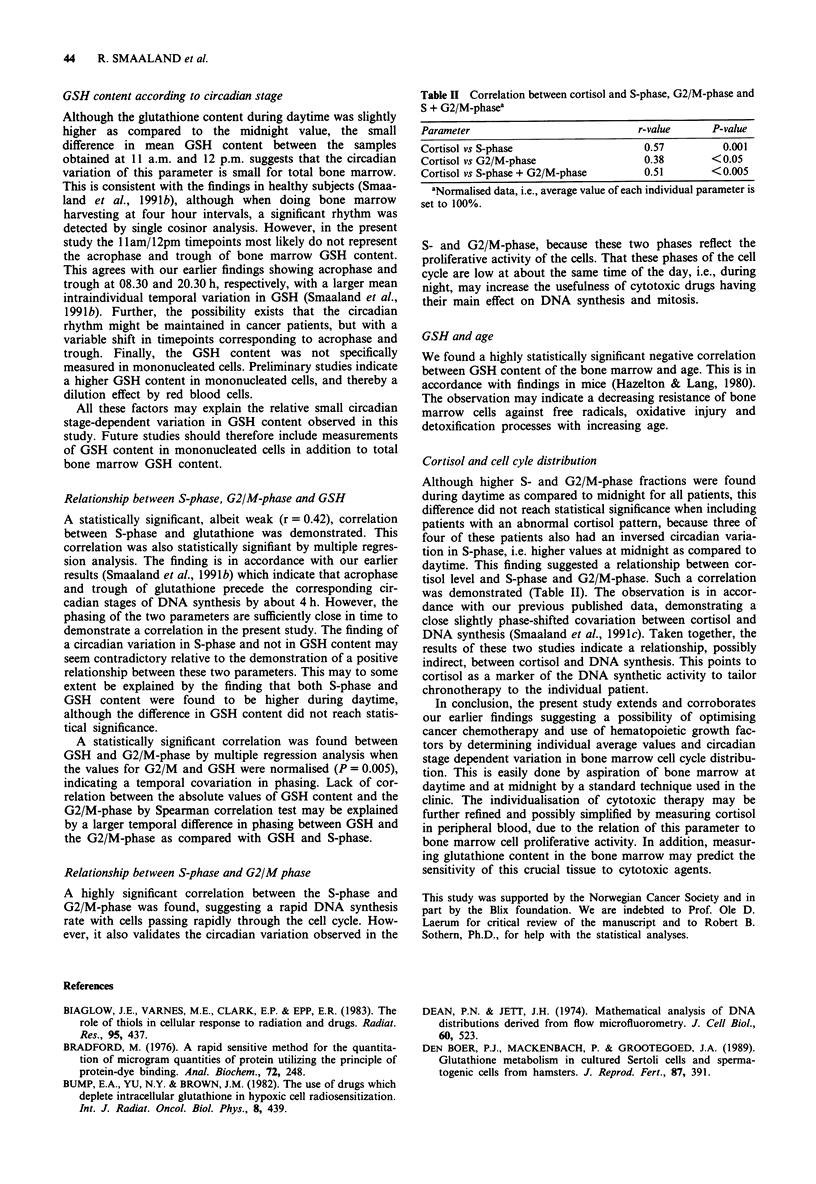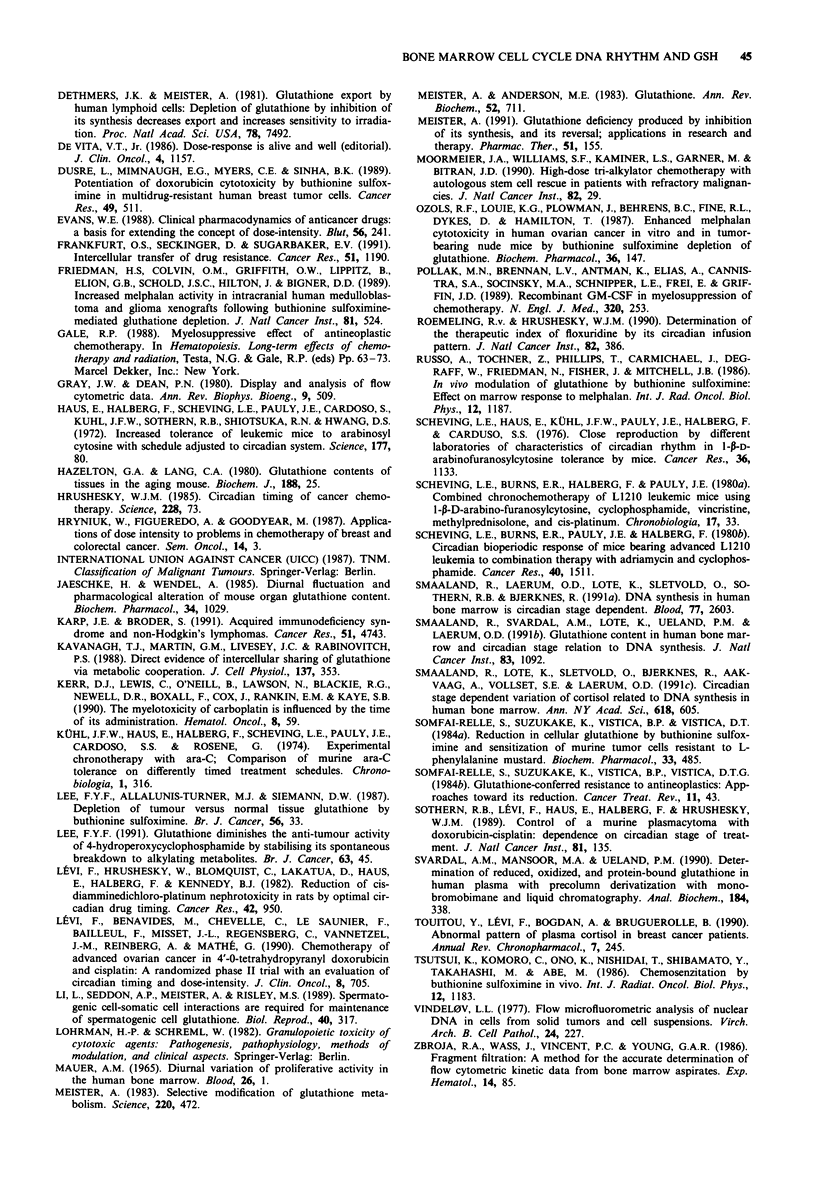# DNA cell cycle distribution and glutathione (GSH) content according to circadian stage in bone marrow of cancer patients.

**DOI:** 10.1038/bjc.1992.213

**Published:** 1992-07

**Authors:** R. Smaaland, J. F. Abrahamsen, A. M. Svardal, K. Lote, P. M. Ueland

**Affiliations:** Gade Institute, Department of Pathology, Haukeland Hospital, University of Bergen, Norway.

## Abstract

DNA cell cycle distribution and glutathione (GSH) content in bone marrow were measured both at daytime and midnight over single 24 h periods in 15 cancer patients. Between patients the S-phase demonstrated a difference from lowest to highest value of 700%, whereas the corresponding difference for the G2/M-phase was nearly 900%. The mean GSH content measured in the bone marrow at the two timepoints was 2.24 +/- 0.21 nmol mg-1 protein, range 0.91-4.19 nmol mg-1 protein. A statistically significant higher fraction of cells in S-phase and G2/M-phase was found at daytime as compared to midnight when excluding the four patients with an abnormal circadian variation in cortisol. No significant temporal variation in total bone marrow GSH content was found, although a weak correlation between S-phase and GSH content was demonstrated (r = 0.42; P less than 0.05). This correlation was strengthened when not including the six patients with an abnormal cortisol pattern (4) and bone marrow infiltration (2) (r = 0.66; P = 0.005). Cells in S-phase demonstrated a positive correlation with cells in G2/M-phase (r = 0.64; P less than 0.0001). A negative correlation was found between GSH content and age (r = 0.53; P less than 0.005). Finally, a statistically significant positive correlation was demonstrated between cortisol and both S-phase and G2/M-phase (r = 0.57; P less than 0.001 and r = 0.38; P less than 0.05, respectively). The present study suggests a possibility of optimising cancer therapy and use of hematopoietic growth factors by determining individual average values and circadian stage dependent variation in bone marrow DNA cell cycle distribution. Furthermore, GSH content in bone marrow may predict this tissue's sensitivity to cytotoxic agents.


*Br. J. Cancer* (1992), **66**, 39–45 © Macmillan Press Ltd., 1992

### DNA cell cycle distribution and glutathione (GSH) content according to circadian stage in bone marrow of cancer patients

R. Smaaland[1,4], J.F. Abrahamsen[2], A.M. Svardal[3], K. Lote[4] & P.M. Ueland[3]

[1]*The Gade Institute, Department of Pathology, Haukeland Hospital, University of Bergen, N-5021 Bergen;* [2]*Division for Geriatric Medicine, Department of Public Health and Primary Health Care, Deaconess Hospital, University of Bergen, N-5009 Bergen;* [3]*Department of Pharmacology and Toxicology, University of Bergen, N-5021 Haukeland Hospital, Bergen;* [4]*Department of Oncology, Haukeland Hospital, University of Bergen, N-5021 Bergen, Norway.*

**Summary** DNA cell cycle distribution and glutathione (GSH) content in bone marrow were measured both at daytime and midnight over single 24 h periods in 15 cancer patients. Between patients the S-phase demonstrated a difference from lowest to highest value of 700%, whereas the corresponding difference for the G2/M-phase was nearly 900%. The mean GSH content measured in the bone marrow at the two timepoints was $2.24 \pm 0.21$ nmol mg$^{-1}$ protein, range 0.91–4.19 nmol mg$^{-1}$ protein. A statistically significant higher fraction of cells in S-phase and G2/M-phase was found at daytime as compared to midnight when excluding the four patients with an abnormal circadian variation in cortisol. No significant temporal variation in total bone marrow GSH content was found, although a weak correlation between S-phase and GSH content was demonstrated ($r = 0.42$; $P < 0.05$). This correlation was strengthened when not including the six patients with an abnormal cortisol pattern (4) and bone marrow infiltration (2) ($r = 0.66$; $P = 0.005$). Cells in S-phase demonstrated a positive correlation with cells in G2/M-phase ($r = 0.64$; $P < 0.0001$). A negative correlation was found between GSH content and age ($r = 0.53$; $P < 0.005$). Finally, a statistically significant positive correlation was demonstrated between cortisol and both S-phase and G2/M-phase ($r = 0.57$; $P < 0.001$ and $r = 0.38$; $P < 0.05$, respectively). The present study suggests a possibility of optimising cancer therapy and use of hematopoietic growth factors by determining individual average values and circadian stage dependent variation in bone marrow DNA cell cycle distribution. Furthermore, GSH content in bone marrow may predict this tissue's sensitivity to cytotoxic agents.

The bone marrow is today the major dose limiting tissue when treating cancer patients with cytotoxic drugs. Bone marrow suppression is generally observed following combination therapy using different anticancer drugs (Evans, 1988; Gale, 1988). It represents a major problem in cancer chemotherapy, since therapeutic response usually requires drug doses inducing bone marrow hypoplasia. Marrow suppression may not only lead to neutropenia and serious infections, but also to dose reductions, postponement of treatment courses and reduced duration of useful treatment. In addition, the possibilities of treatment in the event of relapse may be reduced. This sensitivity to cytotoxic chemotherapy is to a great extent related to the high proliferative activity of bone marrow cells (Lohrman & Schreml, 1982; Pollak *et al.*, 1989), although other factors may contribute as well.

Glutathione (GSH), a cystein-containing tripeptide, has been assigned an important role in the cellular defence against free radicals and reactive oxygen intermediates, as well as in detoxification processes and in the protection of the cell against radiation damage (Dethmers & Meister, 1981; Bump *et al.*, 1982; Meister, 1983; Biaglow *et al.*, 1983; Lee *et al.*, 1987; Friedman *et al.*, 1989). It is the most abundant intracellular non-protein thiol and the cellular content amounts to 0.5–10 nmol l$^{-1}$ (Meister & Anderson, 1983; Dusre *et al.*, 1989). It has been demonstrated that bone marrow of both animals and healthy humans contain a low level of intracellular GSH as compared to other normal tissues (Somfai-Relle *et al.*, 1984*a*; Jaeschke & Wendel, 1985; Tsutsui *et al.*, 1986; Lee *et al.*, 1987; Smaaland *et al.*, 1991*b*), and this may contribute to the reduced tolerance of bone marrow cells towards cytotoxic drugs.

It is well documented that the susceptibility to cancer chemotherapy shows circadian variations in laboratory animals (Haus *et al.*, 1972; Scheving *et al.*, 1976; Lévi *et al.*, 1982). In addition to reduced mortality due to acute toxicity, it has also been shown that an increase in tumour effect or cure rate can be obtained by timing the therapy to periods with less susceptibility of normal cells (Haus *et al.*, 1972; Kühl *et al.*, 1974; Scheving *et al.*, 1980*a*; Scheving *et al.*, 1980*b*; Sothern *et al.*, 1989; Roemeling & Hrushesky, 1990). Furthermore, clinical studies have demonstrated a circadian stage dependence of bone marrow toxicity induced by cytotoxic drugs. There are less dose reductions, less treatment related complications and less postponements of drug courses when drugs have been administered at certain times of the day (Hrushesky, 1985; Kerr *et al.*, 1990; Lévi *et al.*, 1990). We have suggested that these temporal variations in cytotoxic sensitivity of the bone marrow can be explained by a circadian stage dependent variation in DNA synthesis (S-phase) of bone marrow cells, which is significantly lower during night as compared to daytime (Smaaland *et al.*, 1991*a*). A temporal covariation between DNA synthesis and glutathione content of the bone marrow has been demonstrated as well (Smaaland *et al.*, 1991*b*). These studies were performed in healthy male subjects. The present study was performed in order to find out whether these earlier reported results also are valid in cancer patients in whom the circadian rhythmicity might be disturbed due to the malignant disease.

### Materials and methods

### *Chemicals*

N-Ethylmaleimide (NEM), N-ethylmorpholine, dithioerythritol, GSH and GSSG were obtained from Sigma Chemical Co, St. Louis, MO, and sodium borohydride was from Fluka Chemie AG, Switzerland. Dimethylsulfoxide (DMSO), hydrogen bromide (HBr), 5-sulfosalicylic acid (dihydrate), perchloric acid, acetic acid and methanol (for chromatography) were purchased from Merck AG, Dermstadt, F.R.G, and monobromobimane was from Calbiochem, Behring Diagnostics, La Jolla, CA, or Molecular Probes, Eugene, OR.

### *Patients*

From November 1988 to April 1990, 15 patients hospitalised for a malignant disease entered a protocol to study circadian stage variations in bone marrow cell kinetics and glutathione

Correspondence: R. Smaaland.
Received 9 September 1991; and in revised form 9 March 1992.

content. The patients had given their informed written consent to enter the study, which was approved and performed in accordance with the guidelines of the regional medical ethics committee.

All patients were staged according to the 1987 UICC classification (UICC, 1987). One of the patients (UF, M + disease) underwent a painful lymph node biopsy between the bone marrow harvestings. Otherwise the rest of the patients had not suffered any particular physical stress during the last 24 h before bone marrow sampling. Two patients with M + breast carcinoma (MH and SK) had received intensive chemotherphy during the years before the bone marrow harvesting, while the other 13 patients had not received any antineoplastic treatment. Patient characteristics are given in Table I. Nine had stage IV or M + disease and two (SH and NAM) had cytologically verified significant infiltration of malignant cells in the bone marrow. All performance status stages (WHO) are represented. All patients had a regular diurnal rhythm for at least 3 weeks before the bone marrow harvesting. They followed the hospital routine during the study period, except during the two sampling periods. Their biological diurnal rhythm was assessed by measuring the cortisol level at the times of sampling.

Bone marrow cell cycle distribution, measured by flow cytometry, were determined at daytime and at midnight in 15 patients (mean age = 48.7 years; range 27–70 years), i.e. 30 samples, while the glutathione (GSH) content was determined in 14 patients (mean age 49.4 years; range 27–70 years); i.e. 28 samples. One bone marrow sample was lost for GSH content measurement.

*Protocol*

Bone marrow DNA was sampled at 11 a.m. and 12 p.m. The first time of sampling (before noon) was chosen because cytotoxic drugs were usually administered at around 11 a.m. in the hospital. In addition, high DNA synthesis is found in bone marrow cells at this timepoint (Smaaland *et al.*, 1991*a*). The sampling of bone marrow at 12 p.m., i.e. at midnight, was chosen because this time is close to the circadian stage at which the lowest proliferative activity is expected.

*Procedure for bone marrow sampling and sample handling*

The bone marrow was harvested by puncturing the sternum at both timepoints. The sternum was chosen because we have demonstrated a higher DNA synthesis in samples harvested from sternum as compared to the iliac crests (Smaaland *et al.*, 1991*a*).

Bone marrow samples for flow cytometric analysis and GSH content determination were handled and processed as earlier described (Smaaland *et al.*, 1991*a*; Smaaland *et al.*, 1991*b*), one part being immediately put into liquid nitrogen for later GSH content analysis, while another part was stained for flow cytometric analysis according to the method described by Vindeløv (Vindeløv, 1977). Each sample obtained consisted of only 0.2 ml bone marrow in order to avoid peripheral blood admixture and to reduce intra- and interpatient variation in the number of mononucleated cells obtained. Two droplets of each bone marrow sample were used for GSH content determination.

In order to exclude that variations could be attributed to dilution of the samples, caused by local bleeding at the puncture site, differential counts were performed on smears from all individual samples. All smears were characteristic of bone marrow (results not shown).

*Flow cytometry*

The single cell suspension was analysed on a Cytofluorograf 50H, interfaced to a Model 2150 Computer (Ortho Diagnostic Systems, Inc., Westwood, MA, USA). In the cytogram obtained, both peak and area of the fluorescence signal were used for region-setting to discriminate the G1/G0 doublets from the real G2/M cells. Thus, the second peak of the DNA histogram contained only the G2/M cell population. The total number of cells analysed for each sample was $3-4 \times 10^4$. Computerised analyses of the cell cycle distribution in the histograms were done using the constant function of the cell cycle analysis program, by which the percentages of cells in the G1/G0-, S- and G2/M-phases were calculated (Dean & Jett, 1974; Gray & Dean, 1980). The mean CV (coefficient of variation) of the DNA histograms was $2.9 \pm 0.09\%$ (range 2.1–4.0%).

*Determination of reduced (GSH) and oxidised (GSSG + GSSR) glutathione*

A critical step in the determination of glutathione and its different forms is the time between tissue sampling and analysis or freezing in liquid nitrogen. We have found that analysis of bone marrow aspirate which has been instantly frozen in liquid nitrogen and stored either in this medium or at − 80°C gives essentially the same values as an immediate analysis of a fresh aspirate.

The two droplets of bone marrow (in liquid nitrogen) were extracted within 3 days after sampling with 1 ml of ice-cold 5% sulfosalicylic acid containing 50 $\mu$M DTE, and the precipitated protein removed by centrifugation. This protein precipitate was used for subsequent protein determination. GSH and GSSG (oxidised glutathione) + GSSR (soluble glutathione mixed disulfide) were determined in the acid extract by a previously published method (Svardal *et al.*, 1990). Measurement of the bone marrow GSH content was routinely performed in duplicates. The GSH content values presented are the mean value of these two parallel determinations.

*Determination of protein*

The acid precipitated protein was dissolved as previously described (Smaaland *et al.*, 1991*b*) and determined according to Bradford (Bradford, 1976) using the Bio-Rad Protein Assay Kit. Bovine globulin was used as protein standard.

*Statistical analysis*

Analyses were performed on absolute values as well as normalised values, i.e. when the data were expressed as percentage of the individual average value. This procedure was used due to the large interindividual differences in several of the parameters. Data were analysed by Student's *t*-test (paired, two-tailed). Spearman rank correlation coefficients were computed and tests were done to determine whether the *r*-value differed from zero. In addition, multiple regression analyses were performed.

### Results

*Circadian variation in serum cortisol*

Determination of serum cortisol sampled immediately before bone marrow harvesting demonstrated a normal cortisol pattern in 11 of 15 patients, i.e. higher levels during early day (before noon) ($580.7 \pm 73.5$ nmol $l^{-1}$) as compared to midnight levels ($245.2 \pm 27.6$ nmol $l^{-1}$). Three of these 11 patients had stage IV lymphomas, one had M + breast carcinoma, and one had M + malignant melanoma. Of the four patients having an abolished or inversed circadian cortisol rhythm (early day: $377.3 \pm 53.7$ nmol $l^{-1}$; midnight: $424.0 \pm 75.9$ nmol $l^{-1}$), two had stage IV lymphomas, the other two had M + carcinomas, suggesting an abolished internal biological rhythm due to their illness (Table I).

*Cell cycle distribution*

Fractions of cells in G0/G1-phase, S-phase and G2/M-phase were measured at early daytime and at midnight during one 24 h period in 15 patients (30 bone marrow samples). A

**Table I** Clinical patient characteristics, cortisol level and comparison of S-phase, G2/M-phase and GSH content for the two times of bone marrow harvesting

| *Patient* | *Sex* | *Age (years)* | *Malignant disease* | *Stage* | *Perf. st. WHO* | *BMI* | *Cortisol (nmol l)$^{-1}$ 11 a.m.–12 p.m.* | *S-phase (%) 11 a.m.–12 p.m.* | *G2/M-phase (%) 11 a.m.–12 p.m.* | *GSH (nmol mg$^{-1}$ protein) 11 a.m.–12 p.m.* |
|---|---|---|---|---|---|---|---|---|---|---|
| IØ | F | 39 | Hodgkin's disease | IIA | 1 | No | 391–364 | 15.2–10.0 | 2.5–1.2 | 1.36–1.46 |
| AJ | M | 61 | Hodgkin's disease | IVB | 3 | No | 537–288 | 14.8–14.6 | 1.3–0.9 | 2.57–1.52 |
| NPJ | M | 65 | NHL high grade | IIA | 1 | No | 903–203 | 11.3–10.8 | 3.0–2.8 | 0.88–0.94 |
| BT | M | 49 | Malignant melanoma | M + | 1 | No | 610–201 | 17.5–10.2 | 1.7–0.7 | 0.98–1.00 |
| AR | M | 32 | Plasmacytoma | IE | 1 | No | 352–155 | 17.9–12.0 | 3.4–1.7 | 1.95–1.39 |
| IRA | M | 47 | NHL high grade | IIA | 3 | No | 487–228 | 16.2–9.8 | 1.5–1.1 | 2.47–2.32 |
| LBH | M | 27 | Hodgkin's disease | IIIB | 1 | No | 763–231 | 29.1–20.4 | 3.4–3.1 | 4.11–4.27 |
| SS | F | 70 | Adrenal gland carcinoma | M0 | 1 | No | 505–95 | 7.6–5.9 | 0.4–0.5 | 1.31–1.30 |
| MH | F | 56 | Breast carcinoma | M + | 3 | No | 1092–393 | 10.1–3.6 | 1.0–0.5 | 2.02–1.80 |
| UF | F | 48 | Undiff. carcinoma | M + | 2 | No | 223–218 | 11.1–19.1 | 1.8–2.1 | 1.92–2.69 |
| GM | M | 69 | NHL high grade | IVB | 2 | No | 409–460 | 19.9–12.9 | 3.9–2.4 | 2.84–1.80 |
| SAA | M | 27 | Hodgkin's disease | IVB | 1 | No | 405–435 | 10.7–12.7 | 1.1–1.2 | 3.86–3.96 |
| SK | F | 65 | Breast carcinoma | M + | 3 | No | 472–583 | 11.6–19.6 | 1.2–1.2 | 2.91–2.61 |
| NAM | M | 37 | NHL high grade | IVA | 1 | Yes | 426–339 | 11.4–11.3 | 1.1–1.5 | |
| SH | F | 39 | NHL high grade | IVA | 1 | Yes | 322–200 | 8.6–10.4 | 1.9–2.4 | 3.15–3.60 |

NHL = Non-Hodgkin's lymphoma. Perf. st. = Performance status. BMI = Bone marrow infiltration.

considerable interindividual variation in cell cycle distribution was demonstrated. Compared to the lowest individual S-phase and G2/M-phase values, the highest individual values of these two parameters were 700% and 900% larger, respectively.

The lowest and highest individual average S-phase value for the two timepoints was 6.8% and 24.8%. The total mean was 13.2 ± 1.1%.

Correspondingly, the lowest and highest individual average G2/M-phase value for the two timepoints were 0.5% and 3.3%, while the total mean was 1.8 ± 0.2%.

*Glutathione content*

Both reduced and oxidised glutathione in the bone marrow samples were measured. We found that the reduced form of glutathione accounted for 84% of total glutathione, i.e. reduced plus oxidised form.

The mean GSH content measured in the bone marrow of 14 patients (28 samples) at the two timpoints was 2.24 ± 0.21 nmol mg$^{-1}$ protein. The lowest and highest individual average values of the two timepoint measurements were 0.91 nmol mg$^{-1}$ protein and 4.19 nmol mg$^{-1}$ protein, respectively, i.e. a difference of 360%, or about a 3.5 fold difference as compared to the lowest value. The difference between the lowest and highest measured GSH content in each individual ranged from 0.01 nmol mg$^{-1}$ protein to 1.05 nmol mg$^{-1}$ protein, i.e. a significantly smaller absolute variation. In percent the corresponding range of change from lowest to highest GSH content varied from 0.8% to 69.1% as compared to the lowest value, with a mean difference of 19.4%.

*Variation in cell cycle distribution according to circadian stage*

Due to cytologically verified bone marrow infiltration of malignant cells, two patients (NAM and SH) were excluded when analysing a possible circadian stage dependent cell cycle distribution. The remaining 13 patients showed a trend towards higher values of cells in S-phase at daytime as compared to midnight, 14.8 ± 1.4% *vs.* 12.4 ± 1.4%. The same was found for the G2/M-phase, 2.0 ± 0.3 *vs* 1.5 ± 0.2. These differences were not statistically significant ($P = 0.13$ and $P = 0.15$, respectively).

However, when excluding the four patients with an abnormal circadian variation in cortisol, a statistically significant higher fraction of cells in S-phase was found for bone marrow cells harvested at daytime as compared to midnight, whether patients with bone marrow infiltration were included or not (Figure 1). Similar findings were demonstrated for cells in G2/M-phase, with a higher percentage of cells in G2/M-phase at 11 a.m. as compared to 12 p.m. When comparing the proliferative index (S-phase + G2/M-phase, i.e., a measure of the total proliferative activity) between the two timepoints of the circadian scale, the difference between cells harvested at day and midnight was found statistically significant as well (Figure 1). Accordingly, the percentage of non-proliferating cells in G0/G1 were found to be lower during daytime as compared to midnight.

*Glutathione content according to circadian stage*

Although a slightly higher GSH content was found in samples harvested at daytime (2.31 ± 0.28 nmol mg$^{-1}$ protein) as compared to midnight (2.19 ± 0.29 nmol mg$^{-1}$ protein), this difference was not statistically significant ($P = 0.39$). Neither could any significant difference be found when patients with bone marrow infiltration and abnormal circadian cortisol variation were excluded ($P = 0.20$).

*Relation between S-phase, G2/M-phase and glutathione content*

When correlating fraction of cells in S-phase with corresponding values of GSH content, a weak, although statistically significant, correlation was found ($r = 0.42$; $P = 0.028$). A

**Figure 1** Cell cycle distribution (G0/G1-, S-, G2/M- and S- + G2/M-phase) according to circadian stage (11 a.m. *vs* 12 p.m.) for patients with normal cortisol pattern and no bone marrow infiltration. The circadian variation in S-phase of bone marrow cells of healthy subjects when sampled every 4 h (previously published results, see text) is included in the graph to demonstrate the corresponding findings in these two studies.

better relationship was demonstrated when not including patients with an abnormal cortisol pattern and bone marrow infiltration ($r = 0.66$; $P = 0.005$). The two patients with bone marrow infiltration were excluded because tumour cells generally have a higher GSH content as compared to normal cells. Furthermore, by multiple regression analysis a significant correlation was found between S-phase and GSH content ($P = 0.017$).

No correlation was found between the absolute values of G2/M-phase and GSH ($P = 0.22$), nor when not including patients with an abnormal cortisol pattern or bone marrow infiltration ($P = 0.12$). However, a statistically significant correlation was found between these two parameters ($P = 0.006$), also by multiple regression analysis ($P = 0.005$), when the values for G2/M and GSH content were normalised.

### *Relation between S-phase and G2/M-phase*

Both S-phase and G2/M-phase are a measure of actively proliferating cells. When correlating fraction of bone marrow cells in S-phase with the corresponding cells in G2/M-phase, a statistically significant relation was found for absolute values ($P < 0.0001$) (Figure 2), as well as for percent of mean units ($P < 0.001$). However, when performing a mutliple regression analysis the relation did not reach statistical significance for absolute values, but was highly statistically significant when the data were analysed as percent of mean ($P < 0.0001$).

### *Glutathione content and age*

Since two samples were obtained for each patient ($n = 14$), this gave the possibility of comparing 28 paired age-GSH content values. A negative correlation was demonstrated ($r = 0.53$, $P < 0.005$), indicating decreasing GSH content in human bone marrow with increasing age (Figure 3). This correlation was also highly statistically significant by multiple regression analysis ($P = 0.001$).

### *Relation between S-phase, G2/M-phase and cortisol*

It was of interest to relate cortisol, a parameter with a large circadian amplitude and measurable in peripheral blood, to bone marrow cell proliferative activity. Due to large inter-individual differences in cortisol, S-phase- and G2/M-phase values, all values were normalised and expressed as percent

**Figure 2** Correlation between DNA synthesis (S-phase) and G2/mitosis (G2/M-phase) for 15 patients (30 bone marrow samples). $r = 0.64$; $P < 0.0001$.

**Figure 3** Correlation between glutathione content and age in 14 patients (28 bone marrow samples). $r = 0.53$; $P < 0.005$.

of the individual average value. A statistically significant positive correlation was demonstrated between cortisol and S-phase ($P < 0.001$) and G2/M-phase ($P = 0.012$). These correlations were statistically significant by multiple regression as well ($P < 0.0005$ and $P = 0.01$, respectively).

### Discussion

The efficacy of most antineoplastic drugs is dose intensity-dependent, i.e., higher doses over a shorter time span increase the response rates and proportion of cures (Moormeier *et al.*, 1990). Any compromise of dosage or delays in treatment schedule will diminish the likelihood of cancer control or cure (DeVita, 1986; Hryniuk *et al.*, 1987; Evans, 1988). It therefore becomes important to reduce the toxic effects to normal sensitive tissues, especially the bone marrow.

The present study extends our earlier findings in healthy men of a circadian stage dependent variation in DNA synthesis (Smaaland *et al.*, 1991*a*) and glutathione content (Smaaland *et al.*, 1991*b*) in human bone marrow. Based on the results of these studies, we chose to perform the bone marrow sampling at daytime and at midnight, i.e. at times of presumed high and low proliferative activity, respectively. Cortisol level was determined to see whether the endogeneous rhythm of the cancer patients was preserved.

Based on measurement of cortisol pattern we found that five of nine patients with advanced or disseminated disease had a preserved endogeneous rhythm. This is consistent with earlier reported data (Touitou *et al.*, 1990).

A wide range in values of the different phases of the cell cycle was found between the patients, and the variation was even larger than found in DNA synthesis of healthy subjects (Smaaland *et al.*, 1991*a*). This could not be explained by differences in proliferative activity between treated and non-treated patients. These interindividual differences in cell cycle distribution may explain the clinically observed variations in bone marrow sensitivity to cytotoxic drugs in cancer patients. The mean value in S-phase of 13.2% is the same mean value as was found for healthy volunteers (Smaaland *et al.*, 1991*a*). The mean S-phase value of 14.2% for the samples obtained at daytime are also close to values reported for S-phase values obtained by trephine (15.3%) and from filtered bone marrow fragments (16.5%) (Zbroja *et al.*, 1986). These corresponding values of S-phase indicate that dilution of the bone marrow samples with peripheral blood in the present study do not cause falsely low DNA synthesis values. In addition, they validate the differences found in cell cycle distribution at each circadian stage.

### *Bone marrow GSH content*

The total mean GSH content measured was somewhat lower than the previously reported GSH content in healthy individuals ($2.24\ \text{nmol mg}^{-1}$ protein *vs* $2.54\ \text{nmol mg}^{-1}$ protein) (Smaaland *et al.*, 1991*b*). This was not due to a low content of GSH in the two patients having undergone intensive chemotherapy (mean GSH content of these two patients was $2.34\ \text{nmol mg}^{-1}$ protein). Thus, the earlier reported low level of glutathione content in human bone marrow was confirmed (Smaaland *et al.*, 1991*b*), indicating that a low detoxifying capacity may be an explanation for the high sensitivity of the bone marrow to cytotoxic drugs, in addition to the high proliferative activity. The suggested differential response of bone marrow as compared to malignant tumours to pretreatment with the GSH-depleting agent BSO (Smaaland *et al.*, 1991*b*), may indicate a way of preferentially protecting the bone marrow to cytotoxic therapy. This hypothesis is supported by several studies (Somfai-Relle *et al.*, 1984*b*; Russo *et al.*, 1986; Tsutsui *et al.*, 1986; Ozols *et al.*, 1987; Lee, 1991).

Conceivably, admixture of peripheral blood derived red blood cells (RBCs) could contribute to the GSH content measured. By always using the same procedure in bone marrow harvesting, in addition to the small bone marrow sample harvested, we tried to minimise this potential problem. However, we decided to measure GSH content in total bone marrow for two reasons. First, a procedure including a density gradient step to purify the bone marrow cells may itself affect cellular reduced glutathione content. Second, intercellular transport of GSH as a mechanism of transfer of drug resistance between adjacent cells has recently been described (Li *et al.*, 1989; Den Boer *et al.*, 1989; Kavanagh *et al.*, 1988; Frankfurt *et al.*, 1991; Meister, 1991) and GSH content in crude bone marrow may therefore reflect the detoxifying capacity of the bone marrow *in vivo*.

The finding of a 3.5 fold difference between the lowest and highest average GSH content in bone marrow of cancer patients is even larger than reported in our earlier study of healthy subjects (Smaaland *et al.*, 1991*b*). The findings reported in the present study underscore the earlier results of a relative large interindividual difference in bone marrow GSH content, which may indicate differing susceptibility of individual patients to cytotoxic therapy. This may have prognostic relevance in decision making relative to dose intensity when administering cytotoxic drugs to patients.

### *Circadian variation in cell cycle distribution*

A statistically significant higher S-phase and G2/M-phase was found for bone marrow cells harvested during early daytime as compared to midnight for patients with a normal circadian cortisol pattern. This is consistent with our earlier results (Smaaland *et al.*, 1991*a*) and the data of Mauer (Mauer, 1965) who found that percentage of $^{3}$H-TdR labelled cells of the myeloid lineage was higher during the day as compared to midnight in three of four individuals.

Therefore, by taking circadian stage dependent variations of bone marrow cell proliferative activity into account, it may be possible to reduce bone marrow toxicity of S- and G2/M-phase specific drugs or drugs having a major effect on these phases. This may be done by administering the drugs or the major dose of a continuous drug infusion during the time of lowest proliferative activity, i.e. late evening or at night in diurnally-active individuals. Cells in the S- and G2/M-phase will then be less susceptible, and cells in the G1-phase will have more time to repair DNA breaks before entering S-phase and replicating (Karp & Broder, 1991). However, pharmacokinetic and pharmacodynamic properties of the actual cytotoxic drugs must be taken into consideration.

An additional important aspect of the present study as well as the earlier reported findings in healthy subjects, is that it may be possible to increase the effect of biological response modifiers like G-CSF, GM-CSF and IL-3 (Smaaland *et al.*, 1991*a*). This may be done by administering the optimal dose at the time of greatest responsiveness of the bone marrow cells and thereby increase their effect, and possibly also reduce their side effects. These data further suggest that it may be possible to increase the fraction of proliferating cells with careful selection of time of day for harvesting bone marrow cells for auto- or allografting.

*GSH content according to circadian stage*

Although the glutathione content during daytime was slightly higher as compared to the midnight value, the small difference in mean GSH content between the samples obtained at 11 a.m. and 12 p.m. suggests that the circadian variation of this parameter is small for total bone marrow. This is consistent with the findings in healthy subjects (Smaaland *et al.*, 1991*b*), although when doing bone marrow harvesting at four hour intervals, a significant rhythm was detected by single cosinor analysis. However, in the present study the 11am/12pm timepoints most likely do not represent the acrophase and trough of bone marrow GSH content. This agrees with our earlier findings showing acrophase and trough at 08.30 and 20.30 h, respectively, with a larger mean intraindividual temporal variation in GSH (Smaaland *et al.*, 1991*b*). Further, the possibility exists that the circadian rhythm might be maintained in cancer patients, but with a variable shift in timepoints corresponding to acrophase and trough. Finally, the GSH content was not specifically measured in mononucleated cells. Preliminary studies indicate a higher GSH content in mononucleated cells, and thereby a dilution effect by red blood cells.

All these factors may explain the relative small circadian stage-dependent variation in GSH content observed in this study. Future studies should therefore include measurements of GSH content in mononucleated cells in addition to total bone marrow GSH content.

*Relationship between S-phase, G2/M-phase and GSH*

A statistically significant, albeit weak ($r = 0.42$), correlation between S-phase and glutathione was demonstrated. This correlation was also statistically signifiant by multiple regression analysis. The finding is in accordance with our earlier results (Smaaland *et al.*, 1991*b*) which indicate that acrophase and trough of glutathione precede the corresponding circadian stages of DNA synthesis by about 4 h. However, the phasing of the two parameters are sufficiently close in time to demonstrate a correlation in the present study. The finding of a circadian variation in S-phase and not in GSH content may seem contradictory relative to the demonstration of a positive relationship between these two parameters. This may to some extent be explained by the finding that both S-phase and GSH content were found to be higher during daytime, although the difference in GSH content did not reach statistical significance.

A statistically significant correlation was found between GSH and G2/M-phase by multiple regression analysis when the values for G2/M and GSH were normalised ($P = 0.005$), indicating a temporal covariation in phasing. Lack of correlation between the absolute values of GSH content and the G2/M-phase by Spearman correlation test may be explained by a larger temporal difference in phasing between GSH and the G2/M-phase as compared with GSH and S-phase.

*Relationship between S-phase and G2/M phase*

A highly significant correlation between the S-phase and G2/M-phase was found, suggesting a rapid DNA synthesis rate with cells passing rapidly through the cell cycle. However, it also validates the circadian variation observed in the S- and G2/M-phase, because these two phases reflect the proliferative activity of the cells. That these phases of the cell cycle are low at about the same time of the day, i.e., during night, may increase the usefulness of cytotoxic drugs having their main effect on DNA synthesis and mitosis.

**Table II** Correlation between cortisol and S-phase, G2/M-phase and S + G2/M-phase[a]

| *Parameter* | *r-value* | *P-value* |
|---|---|---|
| Cortisol *vs* S-phase | 0.57 | 0.001 |
| Cortisol *vs* G2/M-phase | 0.38 | <0.05 |
| Cortisol *vs* S-phase + G2/M-phase | 0.51 | <0.005 |

[a]Normalised data, i.e., average value of each individual parameter is set to 100%.

*GSH and age*

We found a highly statistically significant negative correlation between GSH content of the bone marrow and age. This is in accordance with findings in mice (Hazelton & Lang, 1980). The observation may indicate a decreasing resistance of bone marrow cells against free radicals, oxidative injury and detoxification processes with increasing age.

*Cortisol and cell cyle distribution*

Although higher S- and G2/M-phase fractions were found during daytime as compared to midnight for all patients, this difference did not reach statistical significance when including patients with an abnormal cortisol pattern, because three of four of these patients also had an inversed circadian variation in S-phase, i.e. higher values at midnight as compared to daytime. This finding suggested a relationship between cortisol level and S-phase and G2/M-phase. Such a correlation was demonstrated (Table II). The observation is in accordance with our previous published data, demonstrating a close slightly phase-shifted covariation between cortisol and DNA synthesis (Smaaland *et al.*, 1991*c*). Taken together, the results of these two studies indicate a relationship, possibly indirect, between cortisol and DNA synthesis. This points to cortisol as a marker of the DNA synthetic activity to tailor chronotherapy to the individual patient.

In conclusion, the present study extends and corroborates our earlier findings suggesting a possibility of optimising cancer chemotherapy and use of hematopoietic growth factors by determining individual average values and circadian stage dependent variation in bone marrow cell cycle distribution. This is easily done by aspiration of bone marrow at daytime and at midnight by a standard technique used in the clinic. The individualisation of cytotoxic therapy may be further refined and possibly simplified by measuring cortisol in peripheral blood, due to the relation of this parameter to bone marrow cell proliferative activity. In addition, measuring glutathione content in the bone marrow may predict the sensitivity of this crucial tissue to cytotoxic agents.

This study was supported by the Norwegian Cancer Society and in part by the Blix foundation. We are indebted to Prof. Ole D. Laerum for critical review of the manuscript and to Robert B. Sothern, Ph.D., for help with the statistical analyses.

**References**

BIAGLOW, J.E., VARNES, M.E., CLARK, E.P. & EPP, E.R. (1983). The role of thiols in cellular response to radiation and drugs. *Radiat. Res.*, **95**, 437.

BRADFORD, M. (1976). A rapid sensitive method for the quantitation of microgram quantities of protein utilizing the principle of protein-dye binding. *Anal. Biochem.*, **72**, 248.

BUMP, E.A., YU, N.Y. & BROWN, J.M. (1982). The use of drugs which deplete intracellular glutathione in hypoxic cell radiosensitization. *Int. J. Radiat. Oncol. Biol. Phys.*, **8**, 439.

DEAN, P.N. & JETT, J.H. (1974). Mathematical analysis of DNA distributions derived from flow microfluorometry. *J. Cell Biol.*, **60**, 523.

DEN BOER, P.J., MACKENBACH, P. & GROOTEGOED, J.A. (1989). Glutathione metabolism in cultured Sertoli cells and spermatogenic cells from hamsters. *J. Reprod. Fert.*, **87**, 391.

DETHMERS, J.K. & MEISTER, A. (1981). Glutathione export by human lymphoid cells: Depletion of glutathione by inhibition of its synthesis decreases export and increases sensitivity to irradiation. *Proc. Natl Acad. Sci. USA*, **78**, 7492.

DE VITA, V.T., Jr. (1986). Dose-response is alive and well (editorial). *J. Clin. Oncol.*, **4**, 1157.

DUSRE, L., MIMNAUGH, E.G., MYERS, C.E. & SINHA, B.K. (1989). Potentiation of doxorubicin cytotoxicity by buthionine sulfoximine in multidrug-resistant human breast tumor cells. *Cancer Res.*, **49**, 511.

EVANS, W.E. (1988). Clinical pharmacodynamics of anticancer drugs: a basis for extending the concept of dose-intensity. *Blut*, **56**, 241.

FRANKFURT, O.S., SECKINGER, D. & SUGARBAKER, E.V. (1991). Intercellular transfer of drug resistance. *Cancer Res.*, **51**, 1190.

FRIEDMAN, H.S, COLVIN, O.M., GRIFFITH, O.W., LIPPITZ, B., ELION, G.B., SCHOLD, J.S.C., HILTON, J. & BIGNER, D.D. (1989). Increased melphalan activity in intracranial human medulloblastoma and glioma xenografts following buthionine sulfoximine-mediated gluthatione depletion. *J. Natl Cancer Inst.*, **81**, 524.

GALE, R.P. (1988). Myelosuppressive effect of antineoplastic chemotherapy. In *Hematopoiesis. Long-term effects of chemotherapy and radiation*, Testa, N.G. & Gale, R.P. (eds) Pp. 63–73. Marcel Dekker, Inc.: New York.

GRAY, J.W. & DEAN, P.N. (1980). Display and analysis of flow cytometric data. *Ann. Rev. Biophys. Bioeng.*, **9**, 509.

HAUS, E., HALBERG, F., SCHEVING, L.E., PAULY, J.E., CARDOSO, S., KUHL, J.F.W., SOTHERN, R.B., SHIOTSUKA, R.N. & HWANG, D.S. (1972). Increased tolerance of leukemic mice to arabinosyl cytosine with schedule adjusted to circadian system. *Science*, **177**, 80.

HAZELTON, G.A. & LANG, C.A. (1980). Glutathione contents of tissues in the aging mouse. *Biochem. J.*, **188**, 25.

HRUSHESKY, W.J.M. (1985). Circadian timing of cancer chemotherapy. *Science*, **228**, 73.

HRYNIUK, W., FIGUEREDO, A. & GOODYEAR, M. (1987). Applications of dose intensity to problems in chemotherapy of breast and colorectal cancer. *Sem. Oncol.*, **14**, 3.

INTERNATIONAL UNION AGAINST CANCER (UICC) (1987). TNM. *Classification of Malignant Tumours*. Springer-Verlag: Berlin.

JAESCHKE, H. & WENDEL, A. (1985). Diurnal fluctuation and pharmacological alteration of mouse organ glutathione content. *Biochem. Pharmacol.*, **34**, 1029.

KARP, J.E. & BRODER, S. (1991). Acquired immunodeficiency syndrome and non-Hodgkin's lymphomas. *Cancer Res.*, **51**, 4743.

KAVANAGH, T.J., MARTIN, G.M., LIVESEY, J.C. & RABINOVITCH, P.S. (1988). Direct evidence of intercellular sharing of glutathione via metabolic cooperation. *J. Cell Physiol.*, **137**, 353.

KERR, D.J., LEWIS, C., O'NEILL, B., LAWSON, N., BLACKIE, R.G., NEWELL, D.R., BOXALL, F., COX, J., RANKIN, E.M. & KAYE, S.B. (1990). The myelotoxicity of carboplatin is influenced by the time of its administration. *Hematol. Oncol.*, **8**, 59.

KÜHL, J.F.W., HAUS, E., HALBERG, F., SCHEVING, L.E., PAULY, J.E., CARDOSO, S.S. & ROSENE, G. (1974). Experimental chronotherapy with ara-C; Comparison of murine ara-C tolerance on differently timed treatment schedules. *Chronobiologia*, **1**, 316.

LEE, F.Y.F., ALLALUNIS-TURNER, M.J. & SIEMANN, D.W. (1987). Depletion of tumour versus normal tissue glutathione by buthionine sulfoximine. *Br. J. Cancer*, **56**, 33.

LEE, F.Y.F. (1991). Glutathione diminishes the anti-tumour activity of 4-hydroperoxycyclophosphamide by stabilising its spontaneous breakdown to alkylating metabolites. *Br. J. Cancer*, **63**, 45.

LÉVI, F., HRUSHESKY, W., BLOMQUIST, C., LAKATUA, D., HAUS, E., HALBERG, F. & KENNEDY, B.J. (1982). Reduction of cis-diamminedichloro-platinum nephrotoxicity in rats by optimal circadian drug timing. *Cancer Res.*, **42**, 950.

LÉVI, F., BENAVIDES, M., CHEVELLE, C., LE SAUNIER, F., BAILLEUL, F., MISSET, J.-L., REGENSBERG, C., VANNETZEL, J.-M., REINBERG, A. & MATHÉ, G. (1990). Chemotherapy of advanced ovarian cancer in 4′-0-tetrahydropyranyl doxorubicin and cisplatin: A randomized phase II trial with an evaluation of circadian timing and dose-intensity. *J. Clin. Oncol.*, **8**, 705.

LI, L., SEDDON, A.P., MEISTER, A. & RISLEY, M.S. (1989). Spermatogenic cell-somatic cell interactions are required for maintenance of spermatogenic cell glutathione. *Biol. Reprod.*, **40**, 317.

LOHRMAN, H.-P. & SCHREML, W. (1982). *Granulopoietic toxicity of cytotoxic agents: Pathogenesis, pathophysiology, methods of modulation, and clinical aspects*. Springer-Verlag: Berlin.

MAUER, A.M. (1965). Diurnal variation of proliferative activity in the human bone marrow. *Blood*, **26**, 1.

MEISTER, A. (1983). Selective modification of glutathione metabolism. *Science*, **220**, 472.

MEISTER, A. & ANDERSON, M.E. (1983). Glutathione. *Ann. Rev. Biochem.*, **52**, 711.

MEISTER, A. (1991). Glutathione deficiency produced by inhibition of its synthesis, and its reversal; applications in research and therapy. *Pharmac. Ther.*, **51**, 155.

MOORMEIER, J.A., WILLIAMS, S.F., KAMINER, L.S., GARNER, M. & BITRAN, J.D. (1990). High-dose tri-alkylator chemotherapy with autologous stem cell rescue in patients with refractory malignancies. *J. Natl Cancer Inst.*, **82**, 29.

OZOLS, R.F., LOUIE, K.G., PLOWMAN, J., BEHRENS, B.C., FINE, R.L., DYKES, D. & HAMILTON, T. (1987). Enhanced melphalan cytotoxicity in human ovarian cancer in vitro and in tumor-bearing nude mice by buthionine sulfoximine depletion of glutathione. *Biochem. Pharmacol.*, **36**, 147.

POLLAK, M.N., BRENNAN, L.V., ANTMAN, K., ELIAS, A., CANNISTRA, S.A., SOCINSKY, M.A., SCHNIPPER, L.E., FREI, E. & GRIFFIN, J.D. (1989). Recombinant GM-CSF in myelosuppression of chemotherapy. *N. Engl. J. Med.*, **320**, 253.

ROEMELING, R.v. & HRUSHESKY, W.J.M. (1990). Determination of the therapeutic index of floxuridine by its circadian infusion pattern. *J. Natl Cancer Inst.*, **82**, 386.

RUSSO, A., TOCHNER, Z., PHILLIPS, T., CARMICHAEL, J., DEGRAFF, W., FRIEDMAN, N., FISHER, J. & MITCHELL, J.B. (1986). *In vivo* modulation of glutathione by buthionine sulfoximine: Effect on marrow response to melphalan. *Int. J. Rad. Oncol. Biol. Phys.*, **12**, 1187.

SCHEVING, L.E., HAUS, E., KÜHL, J.F.W., PAULY, J.E., HALBERG, F. & CARDUSO, S.S. (1976). Close reproduction by different laboratories of characteristics of circadian rhythm in 1-β-D-arabinofuranosylcytosine tolerance by mice. *Cancer Res.*, **36**, 1133.

SCHEVING, L.E., BURNS, E.R., HALBERG, F. & PAULY, J.E. (1980*a*). Combined chronochemotherapy of L1210 leukemic mice using 1-β-D-arabino-furanosylcytosine, cyclophosphamide, vincristine, methylprednisolone, and cis-platinum. *Chronobiologia*, **17**, 33.

SCHEVING, L.E., BURNS, E.R., PAULY, J.E. & HALBERG, F. (1980*b*). Circadian bioperiodic response of mice bearing advanced L1210 leukemia to combination therapy with adriamycin and cyclophosphamide. *Cancer Res.*, **40**, 1511.

SMAALAND, R., LAERUM, O.D., LOTE, K., SLETVOLD, O., SOTHERN, R.B. & BJERKNES, R. (1991*a*). DNA synthesis in human bone marrow is circadian stage dependent. *Blood*, **77**, 2603.

SMAALAND, R., SVARDAL, A.M., LOTE, K., UELAND, P.M. & LAERUM, O.D. (1991*b*). Glutathione content in human bone marrow and circadian stage relation to DNA synthesis. *J. Natl Cancer Inst.*, **83**, 1092.

SMAALAND, R., LOTE, K., SLETVOLD, O., BJERKNES, R., AAKVAAG, A., VOLLSET, S.E. & LAERUM, O.D. (1991*c*). Circadian stage dependent variation of cortisol related to DNA synthesis in human bone marrow. *Ann. NY Acad. Sci.*, **618**, 605.

SOMFAI-RELLE, S., SUZUKAKE, K., VISTICA, B.P. & VISTICA, D.T. (1984*a*). Reduction in cellular glutathione by buthionine sulfoximine and sensitization of murine tumor cells resistant to L-phenylalanine mustard. *Biochem. Pharmacol.*, **33**, 485.

SOMFAI-RELLE, S., SUZUKAKE, K., VISTICA, B.P., VISTICA, D.T.G. (1984*b*). Glutathione-conferred resistance to antineoplastics: Approaches toward its reduction. *Cancer Treat. Rev.*, **11**, 43.

SOTHERN, R.B., LÉVI, F., HAUS, E., HALBERG, F. & HRUSHESKY, W.J.M. (1989). Control of a murine plasmacytoma with doxorubicin-cisplatin: dependence on circadian stage of treatment. *J. Natl Cancer Inst.*, **81**, 135.

SVARDAL, A.M., MANSOOR, M.A. & UELAND, P.M. (1990). Determination of reduced, oxidized, and protein-bound glutathione in human plasma with precolumn derivatization with monobromobimane and liquid chromatography. *Anal. Biochem.*, **184**, 338.

TOUITOU, Y., LÉVI, F., BOGDAN, A. & BRUGUEROLLE, B. (1990). Abnormal pattern of plasma cortisol in breast cancer patients. *Annual Rev. Chronopharmacol.*, **7**, 245.

TSUTSUI, K., KOMORO, C., ONO, K., NISHIDAI, T., SHIBAMATO, Y., TAKAHASHI, M. & ABE, M. (1986). Chemosenzitation by buthionine sulfoximine in vivo. *Int. J. Radiat. Oncol. Biol. Phys.*, **12**, 1183.

VINDELØV, L.L. (1977). Flow microfluorometric analysis of nuclear DNA in cells from solid tumors and cell suspensions. *Virch. Arch. B. Cell Pathol.*, **24**, 227.

ZBROJA, R.A., WASS, J., VINCENT, P.C. & YOUNG, G.A.R. (1986). Fragment filtration: A method for the accurate determination of flow cytometric kinetic data from bone marrow aspirates. *Exp. Hematol.*, **14**, 85.